# 2D Correlation Spectroscopy (2DCoS) Analysis of Temperature-Dependent FTIR-ATR Spectra in Branched Polyethyleneimine/TEMPO-Oxidized Cellulose Nano-Fiber Xerogels

**DOI:** 10.3390/polym13040528

**Published:** 2021-02-10

**Authors:** Giuseppe Paladini, Valentina Venuti, Vincenza Crupi, Domenico Majolino, Andrea Fiorati, Carlo Punta

**Affiliations:** 1 Department of Mathematical and Computer Sciences, Physical Sciences and Earth Sciences, University of Messina, Viale Ferdinando Stagno D’Alcontres 31, 98166 Messina, Italy; gpaladini@unime.it (G.P.); dmajolino@unime.it (D.M.); 2 Department of Chemical, Biological, Pharmaceutical and Environmental Sciences, University of Messina, Viale Ferdinando Stagno D’Alcontres 31, 98166 Messina, Italy; vcrupi@unime.it; 3 Department of Chemistry, Materials, and Chemical Engineering, “G. Natta” and INSTM Local Unit, Politecnico di Milano, Piazza Leonardo da Vinci 32, 20133 Milan, Italy; carlo.punta@polimi.it; 4 Istituto di Scienze e Tecnologie Chimiche, “Giulio Natta” (SCITEC), National Research Council-CNR, 20131 Milan, Italy

**Keywords:** cellulose nano-fibers, temperature perturbation, 2D correlation spectroscopy, nanocellulose composites

## Abstract

Fourier transform infrared spectroscopy in attenuated total reflectance geometry (FTIR-ATR), combined with a 2D correlation analysis, was here employed to investigate temperature-induced spectral changes occurring in a particular type of novel cellulosic-based nano-material prepared using 2,2,6,6-tetramethyl-piperidine-1-oxyl (TEMPO) oxidized and ultra-sonicated cellulose nano-fibers (TOUS-CNFs) as three-dimensional scaffolds, and branched polyethyleneimine (bPEI) as cross-linking agent. The aim was to highlight the complex sequential events involving the different functional groups of the polymeric network, as well as to gain insight into the interplay between the amount of bPEI and the resulting sponge-like material, upon increasing temperature. In this framework, synchronous and asynchronous 2D spectra were computed and analyzed in three wavenumber regions (900–1200 cm^−1^, 1500–1700 cm^−1^ and 2680–3780 cm^−1^), where specific vibrational modes of the cellulosic structure fall, and over a T-range between 250 K and 340 K. A step-by-step evolution of the different arrangements of the polymer functional groups was proposed, with particular regard to how the cooperativity degree of inter- and intramolecular hydrogen bonds (HBs) changes upon heating. Information acquired can be useful, in principle, in order to develop a next-generation, T-sensitive novel material to be used for water remediation applications or for drug-delivery nano-vectors.

## 1. Introduction

Nowadays, the development of novel bio-polymeric materials having cellulose as the main component represents a “hot-topic” in different fields of research due to their high availability, biocompatibility and good mechanical properties [1,2,3]. Despite all these suitable characteristics, the use of cellulose in the engineering of advanced scaffolds is strongly limited by its natural tendency to develop hydrogen bonds between hydroxyl groups of a given chain with nearby oxygens, giving rise to highly crystalline and not-soluble structures. In the last few years, several efforts have been devoted to the functionalization of the native cellulose with the aim of improving its solubility, and modifying its physical and chemical characteristics according to specific applications. Well-established approaches include acylation [4], amination [5] and sulfonation [6], that, exploiting the incorporation of specific functional groups into the cellulose network, give birth to chemically-modified cellulosic materials with new advantageous properties. Among them, composite materials made of cellulose derivatives and amine-rich polymers, belonging to the polyethyleneimines family, have gained considerable attention by the scientific community [7].

In this context, cellulose nano-fibers (CNFs) prepared by selective oxidation and ultra-sonication of cotton cellulose, catalyzed by 2,2,6,6-tetramethyl-piperidine-1-oxyl (TEMPO) radical in a NaBr/NaClO oxidizing environment (TOUS-CNFs), are widely employed and investigated [8,9,10,11,12,13,14,15,16,17].

**Figure 1:**
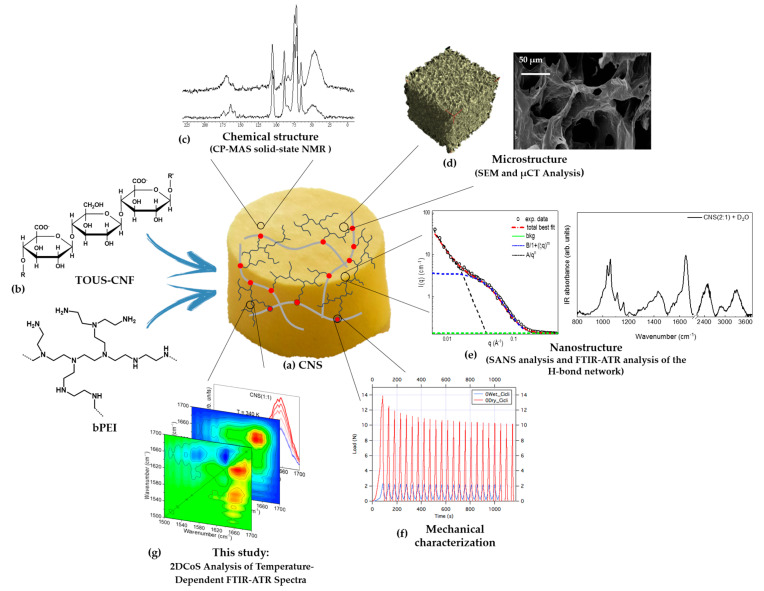
Schematic representation of cellulose nano-sponge (CNS) (**a**). Red dots represent the cross-link points between its polymeric constituents, i.e., TEMPO oxidized and ultra-sonicated cellulose nano-fibers (TOUS-CNFs) and branched polyethyleneimine (bPEI) (grey and black lines, respectively (see panel (**b**)). A detailed characterization was performed, as far as chemical structure (**c**); microstructure (**d**); nanostructure and hydrogen bond (HB) arrangements (**e**); mechanical properties (**f**) are concerned. In this study, 2D Correlation Spectroscopy (2DCoS) Fourier transform infrared spectroscopy in attenuated total reflectance geometry (FTIR-ATR) analysis was exploited for evaluating the effects that temperature can exert on the dynamical properties of the CNS (**g**). See text for details.

Recently, a xerogel (Figure 1a) obtained by the direct cross-linking of TOUS-CNFs with branched polyethyleneimine (bPEI), exploiting the formation of an amidic bond between the carboxylate moieties present on the TOUS-CNFs and the primary amines of bPEI (Figure 1b), was proposed by our research group [18]. The so-obtained material was first characterized by means of cross polarized magic angle spinning (CP-MAS) solid state nuclear magnetic resonance (NMR) (Figure 1c) [18,19], which allowed us to confirm the formation of a stable covalent bond between the two polymers. From a structural point of view, the resulting composites, defined as cellulose nano-sponges (CNSs), possess an interesting sponge-like microstructure which was investigated by means of scanning electron microscopy (SEM) and micro computed tomography (µCT) (Figure 1d) [20]. In addition, a detailed characterization both in terms of structural conformation and dynamics, varying the amount of cross-linker (bPEI) and hydration level, was properly assessed through a combined approach involving small angle neutron scattering (SANS) and Fourier transform infrared spectroscopy in attenuated total reflectance geometry (FTIR-ATR) spectroscopy (Figure 1e) [21,22]. Main results achieved include the observation of nano-sized porosity in the CNS network, as well as the possibility to tailor the dimension of micro/nanopores as a function of the water content. At the same time, a comprehensive and detailed vibrational analysis was carried out on the same samples, revealing a destructuring effect induced by hydration on the hydrogen bond (HB) pattern of interfacial water. Another interesting aspect of CNS is represented by their capability to recover their shape with reduced losses in mechanical resistance, and by the fact that their Young’s modulus can be tuned by varying the CNS formulation (Figure 1f) [19]. Thanks to the chemical moieties (–OH and –NH_x_) present in both constituent polymers, CNS exhibit outstanding features useful for different application fields. First of all, the bPEI amines confer to the composite a remarkable reversible adsorption capability towards heavy metals [18,23,24], organic dyes [25] and drugs [19], exploitable for water remediation processes and drug release. In addition, the exposed functional groups make possible to change the surface charges of the CNS, by simply modifying the pH of the media, allowing the release of the adsorbed metals or organic compounds. It is therefore possible to shift from positive to negative charges, and vice versa [25]. At the same time, by chemical modification of bPEI before the CNS synthesis, new advantageous properties can be obtained. As an example, grafting bPEI with aromatic anhydrides led to the formation of the corresponding mono- and bis-imides, making CNS able to change their color when in presence of fluoride anions, thus acting as selective heterogeneous sensors [20].

In addition, very recently, CNS were proposed as efficient heterogeneous catalysts for the selective synthesis of organic molecules [25]. It was shown how the catalytic performances can be modulated by modifying different parameters, including temperature, achieving results quite different in terms of conversions and products’ distribution, also from those obtained under standard homogeneous conditions at the same temperature. For this reason, an in-deep investigation of the effect of temperature on the inner structure of CNS would become quite important to better understand and exploit the potentialities of this system for this specific purpose. 

The aim of the present work was to evaluate the effects that an external perturbation (i.e., temperature) exerts on the dynamical properties of the CNS system, using a combined approach based on conventional and 2D Correlation Spectroscopy (2DCoS) FTIR-ATR technique. Temperature is expected to affect the dynamics of the bPEI/TOUS-CNF functional groups differently, depending on whether these groups belong to regions with different conformation and mobility of the polymeric mesh, or are internal or external to the nanopores, or come from crystalline/amorphous sections of the system. In this sense, an unambiguous assignment in the measured temperature-dependent FTIR profiles, able to highlight differences in the spectral features induced by one of the aforementioned aspects, turns out to be extremely difficult to be accomplished through conventional 1D analysis, especially in those cases in which complex spectra, consisting of many overlapped peaks, are present. Hence, in order to put into evidence the influence of the polymer structure on the molecular motions, an enhancement of spectral resolution, together with an in-depth analysis of T-induced spectral correlations, is needed. It is worth of note that, on one side, the knowledge of the aforementioned aspects could get an insight into the evolution of the overall CNS inner structure and dynamics upon heating, whose understanding appears extremely useful in the field of heterogeneous catalysis (being both aspects directly related to the different selectivity). On the other side, it could also furnish useful notions for the engineering of temperature-triggered cellulose-based systems for several applications.

In this context, a good strategy consists in describing the molecular dynamics as a function of thermal processing through a 2DCoS analysis of the measured FTIR-ATR profiles (Figure 1g), which allows to estimate the presence of correlations that may exist between perturbation-induced spectral responses, and to properly encode the time sequence of events affecting spectral intensity changes taking place during variation in the external control variable (T) [26,27,28,29,30,31,32,33,34,35,36]. In this work, from the analysis of the 2D spectral intensity changes in the so-called synchronous and asynchronous maps, we propose the sequential order of changes in the molecular environment involving different functional groups. Different 2D patterns were obtained along the same temperature trajectory (250 K–340 K) changing the bPEI: TOUS-CNF weight fraction, once again confirming the strong influence of bPEI on the 3D network of the sponge. 

It is worth remarking that the obtained information could represent a step forward towards the understanding of the complex properties of the investigated CNS material, which could play a central role for a large variety of applications, especially in the field of heterogeneous catalysis.

## 2. Materials and Methods

### 2.1. Synthesis of TEMPO-Oxidized Cellulose and TEMPO Oxidized and Ultra-Sonicated Cellulose Nano-Fibers Dispersion (TOUS-CNFs)

TEMPO oxidized and ultra-sonicated cellulose nano-fibers were obtained as previously reported [17,37].

Briefly, cellulose (10 g) was first dispersed in an aqueous solution (0.57 L) containing potassium bromide (1.54 g, 12.9 mmol) and TEMPO (215 mg, 1.38 mmol), then a NaClO aqueous solution (10% *w/w*, 44 mL, 74 mmol) was added dropwise within 1 h and the mixture was left reacting for 18 h. The pH was maintained around 10.5 by addition dropwise of a NaOH 4 M aqueous solution. After 18 h, the oxidized cellulose pulp was recovered by filtration and then extensively washed with a 1 M HCl aqueous solution (3 × 250 mL) and water (3 × 150 mL). After drying a white powder, composed by TEMPO-oxidized cellulose, was obtained (9.5 g, Y = 95%). The number of carboxylic groups was determined by titration with a NaOH solution, using phenolphthalein as colorimetric indicator.

In order to obtain TEMPO-oxidized and ultra-sonicated cellulose nano-fiber dispersion, the TEMPO-oxidized cellulose was dispersed in water (2.5–3.5% *w/v*) and the suspension was basified by addition of a stoichiometric amount of NaOH. The mixture was ultrasonicated at 0 °C for 30 min (Branson Sonifier 250, Branson Ultrasonic SA, Carouge, Switzerland, 6.5 mm probe tip working at 20 kHz, continuous mode, output power 50%) achieving a gel-like transparent dispersion.

### 2.2. General Procedure for Cellulose Nano-Sponge (CNS) Preparation

CNSs were prepared as previously described in literature [18,19]. In short, TOUS-CNF gel-like dispersion was acidified by means of addition of 2 M HCl solution, up to a pH of about 3. TOUS-CNFs were filtered and washed with deionized water until the eluate reached a pH of about 6/7. The obtained paste-like cellulose mixture was dispersed again in deionized water (3% *w*/*v*), and branched-polyethyleneimine (bPEI, 25 kDa) was added. Table 1 reports the mass ratio between TOUS-CNFs and bPEI used to prepare the samples.

The achieved slurry was sonicated for 10 min obtaining viscous dispersion, which was transferred into molds, frozen and lyophilized for 48 h. In order to form the amidic bond between the carboxylic moieties of TOUS-CNFs and primary amines of bPEI, the resulting xerogels were thermally treated (102 °C, 16 h), achieving a sponge-like material which was extensively washed with methanol. In order to reduce hydration caused by atmospheric humidity, the obtained CNS stored in dry environment. Before any analysis each specimen was grinded in a mortar.

### 2.3. FTIR-ATR Measurements

FTIR-ATR spectra were collected for the three samples in Table 1, in the temperature range extending from T = 250 K to T = 340 K, with a 10-degrees step between each measurement. Measurements were performed in the 400–4000 cm^−1^ wavenumber range, by accumulating 100 repetitive scans in order to guarantee a good signal-to-noise ratio and high reproducibility, with a resolution of 4 cm^−1^. A DA8 Fourier transform infrared spectrometer (BOMEM, Saint Laurent, QC, Canada) was used, operating with a Globar source, a KBr beamsplitter and a thermoelectrically cooled deuterated triglycine sulphate (DTGS) detector. Samples were placed in contact with the surface of a single reflectance ATR cell (Golden Gate, equipped with a diamond crystal). The collected FTIR spectra were properly normalized in order to account for the effective number of absorbers. No smoothing was applied, whereas baseline adjustment and normalization were performed using the Spectracalc software package GRAMS (Galactic Industries, Salem, NH, USA).

### 2.4. 2D Correlation Spectroscopy (2DCoS)

**Scheme 1:**
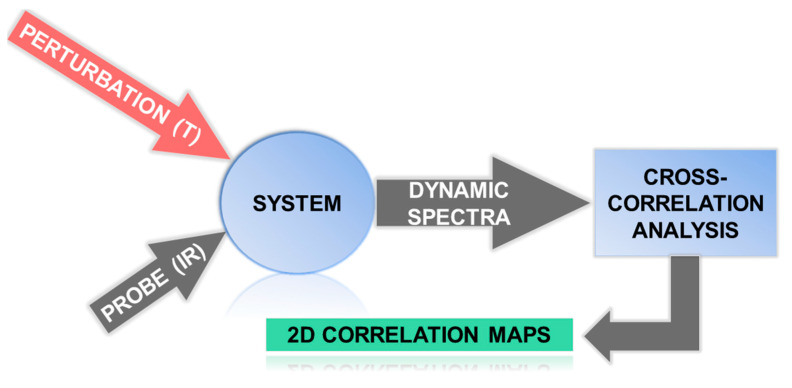
Overall scheme for the evaluation of the 2D correlation maps starting from the FTIR-ATR experimental spectra.

2D FTIR-ATR correlation intensities were computed using the OriginLab^®^ (Northampton, MA, USA) software (ver. 2019) following the generalized 2D correlation criteria developed by Noda [38]. Briefly, a group of perturbation-induced spectra is collected and then transformed in 2D correlation maps through a well-established cross-correlation analysis (see Scheme 1). First, the dynamic FTIR-ATR spectrum ($\tilde{I}\left(\nu ,T\right)$) at a given temperature T was evaluated from the experimental one (*I*(*v*, *T*)) as:

(1) $$\tilde{I}\left(\nu ,T\right)=\{\begin{array}{c} I\left(\nu ,T\right)-\overset{¯}{I}\left(\nu \right)\;for\;T_{min}\leq T\leq T_{max}\\ 0\;\;\;\mathrm{otherwise} \end{array},$$

where $\overset{¯}{I}\left(\nu \right)$ represents a reference spectrum, and *T_min_* and *T_max_* the minimum and maximum values (250 K and 340 K, respectively) assumed by the perturbation variable (temperature in our case). Worthy of note, the reference spectrum in 2DCoS is usually arbitrarily chosen. Commonly, an average or the first/last spectrum of a given series is used, although $\overset{¯}{I}\left(\nu \right)=0$ is also acceptable.

For our calculations, the experimental FTIR-ATR spectrum collected at T = 250 K was chosen as reference, hence $\overset{¯}{I}\left(\nu \right)=I\left(\nu ,T_{min}\right)=I\left(\nu ,250K\right)$. This ensured a proper interpretation of the 2D spectral intensities, providing information about the simultaneous or sequential events occurring in our CNSs upon upward temperature variations. 

According to the theory developed by Noda [38,39,40,41], two different types of 2D correlation spectra can be defined, namely synchronous ($\Theta \left(\nu_{1},\nu_{2}\right)$) and asynchronous ($\Omega \left(\nu_{1},\nu_{2}\right)$), providing different information on the time-dependent order of changes in the molecular behavior. The synchronous and asynchronous 2D maps can be calculated as follows:

(2) $$\Theta \left(\nu_{1},\nu_{2}\right)=\frac{1}{k-1}\overset{k}{\underset{i=1}{\sum }}\tilde{I}\left(\nu_{1},T_{i}\right)\times \tilde{I}\left(\nu_{2},T_{i}\right)$$

(3) $$\Omega \left(\nu_{1},\nu_{2}\right)=\frac{1}{k-1}\overset{k}{\underset{i=1}{\sum }}\tilde{I}\left(\nu_{1},T_{i}\right)\times \left(\overset{k}{\underset{j=1}{\sum }}M_{i,j}\times \tilde{I}\left(\nu_{2},T_{j}\right)\right)$$

where *k* is the number of dynamic spectra collected during the heating process (in our case *k* = 10, see Section 2.3) and *M_i_*_,*j*_ represents the *i*-th row and *j*-th column element of the so-called Hilbert–Noda transformation matrix, defined as:

(4) $$M_{i,j}=\{\begin{array}{c} 0\;\;\;\mathrm{if}\;i=j\\ \frac{1}{\pi \left(j-i\right)}\;\mathrm{otherwise} \end{array}$$

Generally, the synchronous spectrum describes synchronized or coincidental changes of spectral intensities associated to the original dataset, measured at the (*v*_1_, *v*_2_) point of the 2D plane. In other words, peaks on the synchronous map develop only if the band intensities of the experimental spectra at *v*_1_ and *v*_2_ increase or decrease together. The asynchronous spectrum, on the other hand, accounts for unsynchronized events which may arise from delayed or accelerated processes involving correlated vibrational modes upon heating. 

The sign of the observed peaks in both maps is extremely important and deserves some clarifications. If, at a given point (*v*_1_, *v*_2_), results *Θ*(*v*_1_, *v*_2_) > 0 and *Ω*(*v*_1_, *v*_2_) > 0 for the synchronous and asynchronous spectra, respectively, then the corresponding vibrational mode centered at $\nu_{1}$ is affected by the external perturbation prior the one at *v*_2_. An opposite scenario is achieved when *Ω*(*v*_1_, *v*_2_) < 0. On the contrary, if a negative synchronous correlation intensity is observed (*Θ*(*v*_1_, *v*_2_) < 0), the aforementioned rules must be reversed (see Table 2).

It is worth remarking that the aforementioned generalized 2D correlation criteria can be applied for a broad range of spectroscopic probes (IR, Raman, UV-Vis, XRF, NRM, etc.), for any kind of external perturbation (i.e., thermal, chemical, electrical, biological). However, one of the main limitations of the 2DCoS analysis relies in the noise level characterizing the original dataset. In fact, if it exceeds a certain threshold value, the occurrence of artifactual features, or the disappearance of real ones, within the calculated synchronous and asynchronous correlation maps, can be observed. In that case, a noise-reduction pre-treatment becomes mandatory. Going on, the computation procedure used to evaluate the 2D correlation maps described here assumes that raw spectra have been sampled following a constant increment along the external variable (temperature). In this way, a discrete data-set entailing equally spaced spectral traces is obtained. However, in the case of unevenly spaced acquisitions, the 2D correlation analysis cannot be simply accomplished by means of the computational strategy adopted here. Such a limitation can be overcome by properly re-arranging the original computation procedure following the analytical strategy reported in ref. [42] or, equivalently, by conversion of the unevenly spaced data into an equally spaced data-set through interpolation or curve-fitting.

## 3. Results and Discussion

**Figure 2:**
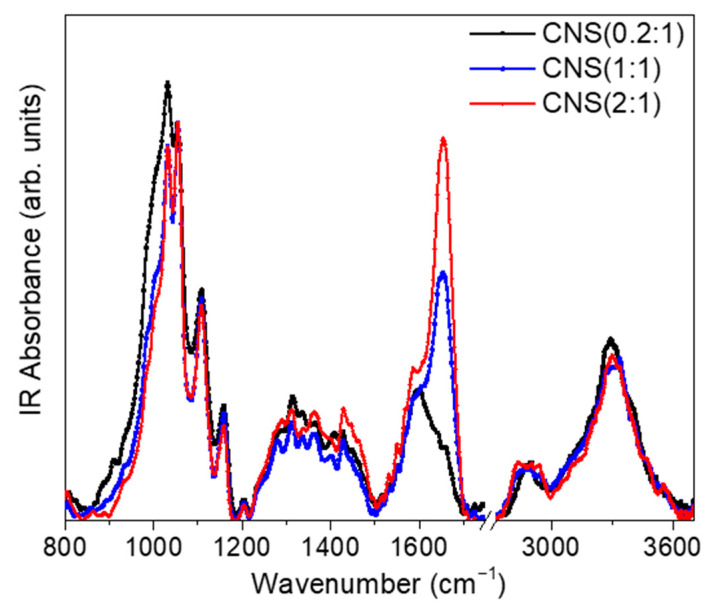
FTIR-ATR spectra in the 800–3700 cm^−1^ wavenumber range, at T = 300 K, of dry CNS (0.2:1) (black line), CNS (1:1) (blue line) and CNS (2:1) (red line) samples.

A proper description of the vibrational modes observed in our materials by FTIR-ATR spectroscopy is necessary before proceeding with the analysis, through 2DCoS, of their dynamical evolution upon heating. Figure 2 depicts the FTIR-ATR spectra of dry CNS (0.2:1), CNS (1:1) and CNS (2:1) samples, at T = 300 K, in the wavenumber range between 800 and 3700 cm^−1^.

As can be seen from the obtained high-quality profiles, all spectra exhibit features whose intensity changed as the amount of bPEI increased, indicating a strong influence of the cross-linker in defining the structure of the resulting cellulose-based sponge-like material. In particular, the fingerprint region revealed bands at ~899 cm^−1^, ~985 cm^−1^, ~1000 cm^−1^, ~1031 cm^−1^, ~1055 cm^−1^, ~1108 cm^−1^, ~1159 cm^−1^ and ~1203 cm^−^^1^, all associated to the cellulosic polymer network [43,44,45,46]. In more detail, bands at ~899 cm^−1^, ~985 cm^−1^, ~1000 cm^−1^, ~1031 cm^−1^ and ~1055 cm^−1^ could be ascribed to the C–O–C stretching vibration of glycoside bonds (~899 cm^−1^), in-plane –CH– rocking (985–1000 cm^−1^), and C–OH stretching mode of the primary (~1031 cm^−1^) and secondary (~1055 cm^−1^) alcohol of cellulose fibers, respectively. The latter, being related to the cellulose backbone, was not affected by TEMPO-mediated oxidation [47,48], and could therefore be considered as an internal standard for quantitative evaluations. Going on, bands at ~1108 cm^−1^ and ~1159 cm^−1^ accounted for the asymmetric C–O–C stretching and C–C breathing mode of cellulose rings. Finally, a low-intensity contribution centred at ~1203 cm^−1^ could be observed, assigned to the C–OH (or C–CH) bending mode. Bands at ~1311 cm^–1^, 1370 and ~1428 cm^–1^ were attributed to CH_2_ rocking vibration of amorphous cellulose, and CH/CH_2_ bending modes, respectively [44,49,50]. In particular, as evident from an inspection of Figure 2, the 1500–1700 cm^−1^ wavenumber region appeared extremely sensitive to the presence of bPEI. This is because vibrations of functional groups directly involved, through the activation of strong covalent interactions, in the growth of the complex CNS network, typically fall in this spectral range. In more detail, bands at ~1583 cm^−1^ at ~1596 cm^−1^, almost overlapped, were assigned to the residual –COO^−^ and N–H deformation modes of the TEMPO-oxidized cellulose fibers and polyethyleneimine, respectively [19,51]. Going on, the strong contribution centered at ~1654 cm^−1^, barely distinguishable in CNS (0.2:1), is due to the formation of amide moieties (–CONH–). The latter, as expected, experienced a significant enhancement in intensity as the amount of bPEI increases, reflecting the presence of more cellulose carboxylic moieties involved in covalent linkage with bPEI. Worthy of note, the absence of the contribution at ~1730 cm^−1^, associated to “free” carboxyl functional groups of cellulose, suggested that almost all the C=O groups were of amidic type. As far as the high-frequency region of the spectra is concerned, the band between ~2750 cm^−1^ and ~3000 cm^−1^ was assigned to the CH and CH_2_ symmetrical stretching arising from both cellulose and bPEI chains. Finally, in the 3000–3700 cm^−1^ wavenumber region, the almost overlapped N–H (~3307 cm^−1^) and O–H (~3330 cm^−1^) stretching vibration modes could be observed. In the latter case, a proper distinction of the possible HB arrangements occurring into the CNS network should be assessed. In fact, according to numerical simulations [52], native cellulose holds eight hydrogen bonds per glucose unit, whereas amorphous cellulose contains 5.3 hydrogen bonds. This discrepancy is at the basis of the different reactivity of crystalline/amorphous regions against external stimuli (i.e., temperature), exhibited by a large variety of cellulose-based materials. Finally, since CNS are highly hygroscopic, O–H stretching vibrations arising from adsorbed water molecules from humid air should be also taken into account, despite the specimens being stored in a dry environment before use.

In our case, by considering the synthetic procedure of CNS, coexistence of a crystalline and an amorphous phase in all the investigated dry functionalized cellulosic samples should be expected. Since crystallinity only depended on cellulose segment orientations and displacements, no variations were likely to occur as the amount of bPEI increased, or within the explored temperature range. However, a reduction of the crystalline phase was expected as a consequence of the synthetic procedure, since it involved treatments with alkaline solutions, which are reported to induce high-ordered (crystalline)/low-ordered (amorphous) fiber transitions [53,54]. In this context, it is worth remarking that similar functional groups belonging to different conformational phases may respond in different way to external perturbations, being involved in different molecular environments. Accordingly, the temperature-dependent molecular dynamics of the resulting CNS supramolecular structure, as evaluated by 2DCoS, will have to take into account whether these functional groups arise from crystalline or amorphous regions. 

**Figure 3:**
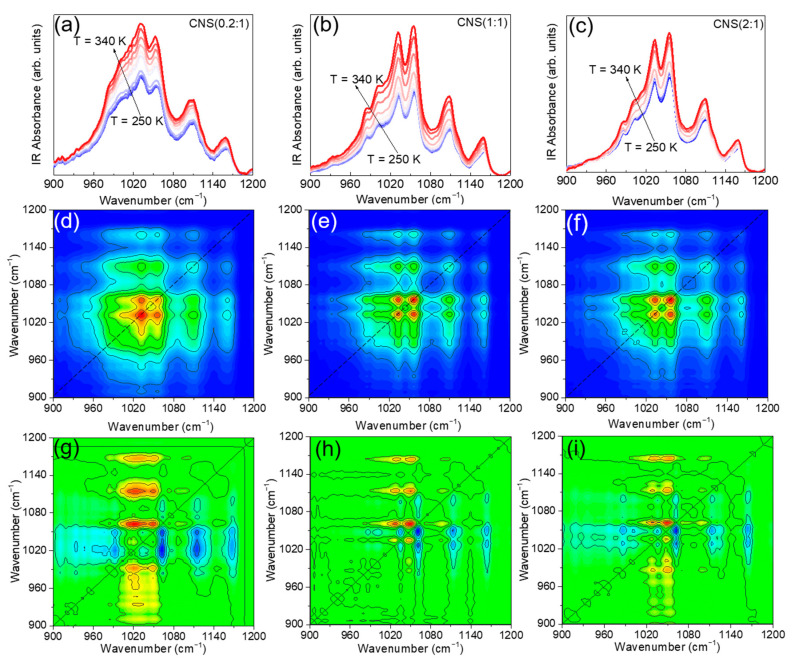
Temperature-dependent FTIR-ATR spectra (**a**–**c**) of the investigated samples in the 900–1200 cm^−1^ wavenumber range; corresponding, synchronous and asynchronous 2D correlation maps (SCMs and ACMs) for CNS (0.2:1) ((**d**) and (**g**), respectively), CNS (1:1) ((**e**) and (**h**), respectively) and CNS (2:1) ((**f**) and (**i**), respectively). In the case of SCMs, red-colored regions define positive correlation intensity (*Θ*(*v*_1_, *v*_2_) > 0), whereas blue-colored areas account for a *Θ*(*v*_1_, *v*_2_) ~ 0 condition. Conversely, in ACMs, red, green and blue-colored areas describe positive, null and negative correlation intensities, respectively. See text for details.

Based on the aforementioned results, synchronous and asynchronous 2D correlation maps (SCMs and ACMs, respectively) were assessed as a result of temperature-dependent spectral changes occurring in the CNS material upon heating, using formulas described in Section 2.4. Figure 3 shows the SCMs and ACMs for all the investigated samples in the 900–1200 cm^−1^ wavenumber range, over the 250 K–340 K temperature range (250 K used as reference). Generally, in the case of SCMs, two types of correlation peaks can be recognized: auto-peaks (APs, always positive) and cross-peaks (CPs, either positive or negative). APs develop only along the diagonal line (*v*_1_ = *v*_2_) and describe the total extent of spectral intensity variation observed at the specific spectral variable *v*. Conversely, CPs are always found in off-diagonal regions and define simultaneous responses of FTIR-ATR signals related to functional groups involved in different molecular environments. 

Before discussing the information regarding the time sequence of events affecting different chemical groups as a function of T, contained in the ACMs, a brief description of the obtained SCMs is reported.

An inspection of Figure 3d–f reveals the presence of two prominent, well-defined APs at ~(1030,1030) cm^−1^ and ~(1055,1055) cm^−1^, and a low-intensity one centered at (1108,1108) cm^−1^, highlighting the changes that simultaneously occurred on the collective ν(C–OH) modes of the primary (~1031 cm^−1^) and secondary (~1055 cm^−1^) alcohol, and on C–C breathing mode of cellulose rings (~1108 cm^−1^) upon heating. All the observed correlation peaks were positive, indicating that temperature-induced variations occurred according to a uniform trend, similarly to what reported in the 1D FTIR-ATR spectra displayed in Figure 3a–c. In addition, by increasing the amount of bPEI, a slight reduction of the AP intensity at ~(1030,1030) cm^−1^ in conjunction with an increase of that at ~(1055,1055) cm^−1^ could be recognized. The high AP intensity at ~(1030,1030) cm^−1^ in CNS (0.2:1) indicated a remarkable susceptibility of this band by thermal motion. Differently, in the case of CNS (1:1) and CNS (2:1), the highest susceptibility was found to be at ~(1055,1055) cm^−1^, presumably as a consequence of changes in the molecular environment triggered by the presence of more amino functional groups of branched polyethyleneimine.

Going on, the possibility of creating a set of correlation “squares” among all the observed CPs and APs suggests that, in the investigated wavenumber region, all modes were strongly entangled with each other, sharing similar temperature dependence, in such an extent determined by the mobility of the corresponding functional group within the polymer network. The onset of a new nearly positive cross-correlation peak at ~(1108,1055) cm^−1^ in CNS (1:1) and CNS (2:1) samples, not detected in CNS (0.2:1), indicated a new coincidental correlation of the corresponding modes, activated by the higher amount of bPEI.

**Figure 4:**
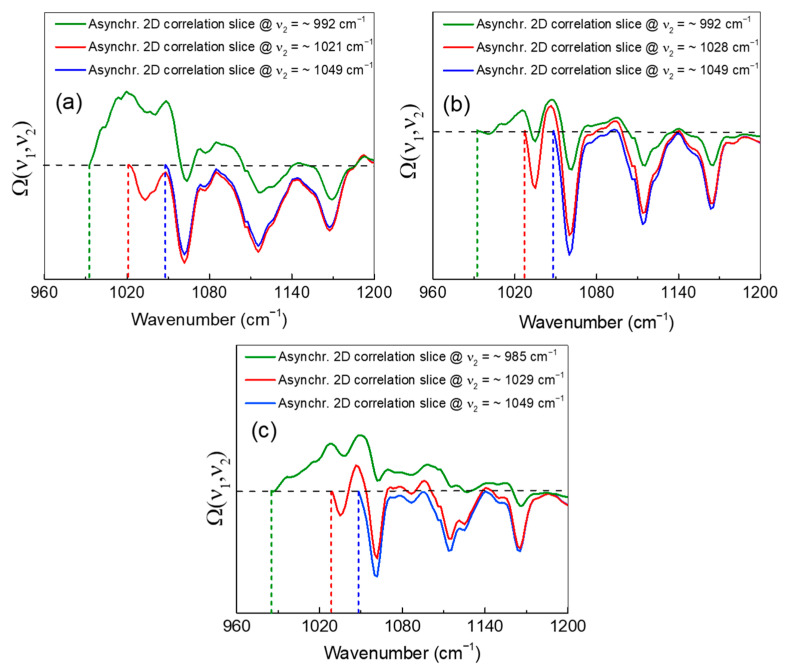
Asynchronous correlation slices at three different values of $\nu_{2}$ for (**a**) CNS (0.2:1), (**b**) CNS (1:1) and (**c**) CNS (2:1) sample. The black dashed line in each panel accounts for the *Θ*(*v*_1_, *v*_2_) ~ 0 condition. Above and below this line, positive and negative correlations can be respectively observed. Being all information regarding the spectral sequence of events contained within the half region of the whole ACM for which *v*_1_ > *v*_2_, the selected slices develop beyond the diagonal line of each map starting from those points for which *v*_1_ = *v*_2_ (colored vertical short-dashed lines).

A completely different scenario emerged by looking at the ACMs in the same region (Figure 3g–i). A first inspection reveals the developing of four-leaf clover patterns, whose position and conformation slightly changed as the amount of bPEI in the CNS network increased. Both positive (red areas) and negative (blue areas) asynchronous correlation peaks could be recognized, suggesting the presence of a not straightforward sequence of “events” involving all CNS modes falling in this spectral range, in response to thermal motion. It is worth underlying that the spreading of the observed experimental FTIR-ATR spectra over the second dimension led to an enhancement of the spectral resolution, that allowed the observation of overlapped contributions otherwise impossible to be revealed by conventional 1D FTIR-ATR spectroscopy. Considering the triangular region of the ACMs for which *v*_1_ > *v*_2_, both negative and positive correlations could be observed. In particular, the presence of localized CPs, all characterized by *v*_2_ ~ 992 cm^−1^, indicated a strong asynchronicity of this vibrational mode with several modes of the bPEI/TOUS-CNFs. The same behavior was clearly visible at *v*_2_ ~ 1021 cm^−1^ and *v*_2_ ~ 1049 cm^−1^. In order to better resolve the sequential order of events affecting, upon heating, the low-frequency modes of the CNS structure, Figure 4 reports the three different horizontal slices obtained from the ACMs at the aforementioned values of *v*_2_.

First of all, as can be observed from both Figure 3g–i and Figure 4, the characteristic correlation patterns were found to be less and less broadened as the amount of bPEI increased. This occurrence testified to the onset of new bPEI-activated linkages between cellulose segments, resulting in a constrained molecular environment. Accordingly, the effect of temperature on the collective modes of the sponge network was expected to be different, due to changes in the strength of the interactions affecting the molecular vibrations.

As far as sample CNS (0.2:1) is concerned, the asynchronous 2D correlation slice at *v*_2_ ~ 992 cm^−1^ (green line of Figure 4a) revealed the presence of two positive correlations with peaks at ~1021 cm^−1^ and ~1053 cm^−1^. According to Noda’s rules and considering the signs of the corresponding SCMs in the same region (always positive), we can state that peaks at ~1021 cm^−1^ and ~1053 cm^−1^ dynamically changed prior the vibrational mode at ~992 cm^−1^ upon an upward temperature trajectory. Noteworthy, the peak at ~1021 cm^−1^, which could be assigned to C–N vibrations of terminal C–NH_2_ units [55,56], was not clearly resolved in 1D FTIR-ATR spectra (see Figure 3a, for example). This sequence showed that the stretching vibrations of C–N groups of terminal units and of C–OH groups of secondary alcohols of amorphous regions in cellulose nano-fibers change their dipole moment first, followed by the C–H bending mode. This occurrence can be explained considering that, the amount of bPEI in sample CNS (0.2:1) being lower than the amount of TOUS-CNFs, few covalent connections among cellulose nano-fibers were likely to occur, resulting in the formation of small aggregates within the polymer network. As a matter of fact, the cross-linking density in such a sample resulted higher with respect to those samples characterized by higher amount of bPEI, for which the development of more cellulose chain–chain connections occurred, giving rise to larger clusters [21]. In other words, more TOUS–CNFs were linked to the same bPEI polymer. This caused a more rigid structure to take place, characterized by polymeric chains having a low degree of freedom, with the exception of those close to terminal sections or within amorphous regions of the reticulated 3D network. According to this scenario, not-constrained groups (C–N of terminal units and C-OH of secondary alcohols) changed their conformation sooner and more easily with respect to hardly-accessible inner groups (C–H). Going on, the slice at *v*_2_ ~ 1021 cm^−1^ (red line of Figure 4a) revealed strong negative cross-correlations of such mode with peaks at ~1030 cm^−1^, ~1060 cm^−1^, ~1110 cm^−1^ and ~1161 cm^−^^1^, highlighting the following sequence of events: C–N vibrational modes of terminal units change first (in agreement with what previously observed), followed by the C–OH stretching of the primary and secondary alcohols of cellulose nano-fibers in amorphous regions [57], and then by the asymmetric C–O–C stretching and C–C breathing mode of cellulose rings. This result could be considered as an experimental proof of the effect that chemical groups mobility exerts on the resulting 3D structure when subjected to temperature perturbation. In particular, the aforementioned sequence suggested that the first conformational change was experienced by almost peripherical sections, reasonably amorphous and hence less ordered with respect to inner cross-linked regions. The amount of energy transferred during the heating process to the C–O–C and C–C breathing modes of cellulose rings was remarkably delayed with respect to the aforementioned process, likely due to the reduced mobility degree of such units. Unfortunately, no further information could be extracted by the observation of the slice at *v*_2_ ~ 1049 cm^−1^ (blue line in Figure 4a), the profile being almost identical to that at *v*_2_ ~ 1021 cm^−1^ highlighting the same sequence of events involving the low-frequency modes of the CNS (0.2:1) structure.

In the case of CNS (1:1) (see Figure 4b), the asynchronous 2D correlation slice at *v*_2_ ~ 992 cm^−1^ indicated weaker correlations with peaks at ~1021 cm^−1^ and ~1053 cm^−1^, contrary to what observed for CNS (0.2:1). The above correlations, on the other side, disappeared in sample CNS (2:1), where positive correlations shifted to the horizontal slice at *v*_2_ ~ 985 cm^−1^ (green line in Figure 4c), revealing a strong influence of cross-linker in regulating the conformational behavior of specific localized functional groups. Interestingly, for both CNS (1:1) and CNS (2:1) samples, asynchronicity was found between the peak at ~1028 cm^−1^, related to the C–OH stretching of the primary alcohol of amorphous cellulose, and modes at ~1060 cm^−1^, ~1110 cm^−1^ and ~1161 cm^−1^. As a result, the first groups to change their conformation upon thermal motion were, in these systems, C–OH groups of CNFs, instead of C–N groups of mostly amorphous terminal units as observed for CNS (0.2:1), suggesting that most of the C–N active sites of the CNS network were involved in amidic covalent interactions.

**Figure 5:**
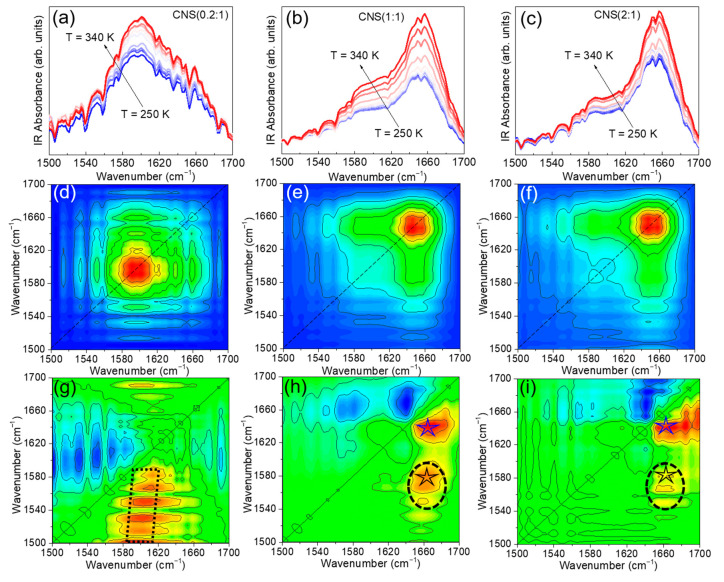
Temperature-dependent FTIR-ATR spectra (**a**–**c**) of the investigated samples in the 1500–1700 cm^−1^ wavenumber range; corresponding SCMs and ACMs for CNS (0.2:1) ((**d**) and (**g**), respectively), CNS (1:1) ((**e**) and (**h**), respectively) and CNS (2:1) ((**f**) and (**i**), respectively). In the case of SCMs, red-colored regions define positive correlation intensity (*Θ*(*v*_1_, *v*_2_) > 0), whereas blue-colored areas account for a *Θ*(*v*_1_, *v*_2_) ~ 0 condition. Conversely, in ACMs, red, green and blue-colored areas describe positive, null and negative correlation intensities, respectively. The black and blue stars in panels (**h**,**i**) show CPs located at ~(1654,1583) cm^−1^ and ~(1654,1635) cm^−1^. Dotted (in (**g**)) and dashed (in (**h**,**i**)) lines highlight regions of interest for our discussion. See text for details.

In Figure 5, the SCMs and ACMs for all the investigated samples in the 1500–1700 cm^−1^ wavenumber range, and over the 250 K–340 K temperature range (250 K used as reference), are displayed.

This region being characteristic of the oxidized cellulose -C=O groups of the carboxylic acid moieties involved in amide bonds with bPEI, knowledge of the temporal sequence affecting these oscillators could furnish, in principle, a deeper understanding of the dynamical behavior exhibited by the entire sponge-like structure upon thermal stimuli. Nevertheless, not all the C=O groups of TEMPO-oxidized CNFs undergo covalent linkages with the amino groups of bPEI [18]. This means that, by increasing temperature, two different dynamical responses are expected, depending on whether the C=O groups are effectively engaged (amide C=O) or not (carboxylic C=O, COOH or –COO^−^) in amidic connections formed during the polymerization process. As can be seen, the SCM calculated for sample CNS (0.2:1) (Figure 5d) shows the presence of an extended positive AP at ~(1596,1596) cm^−1^. This correlation island also embraced the AP at ~(1583,1583) cm^−1^, indicating a strong susceptibility of both the –COO^−^ and N-H groups by temperature. Interestingly, no AP was found at around (1654,1654) cm^−1^. This occurrence can be interpreted by considering that, the amount of primary amino groups of bPEI involved in amide covalent interactions with cellulose nano-fibers being reasonably low for this sample, the effect of temperature on such vibrational mode is not observable. By looking at Figure 5e,f, a shift of the observed pattern from ~(1596,1596) cm^−1^ to ~(1654,1654) cm^−1^ for CNS (1:1) and CNS (2:1) can be clearly observed. This reflects the onset of temperature-sensitive amide moieties (–CONH–) induced by the increased amount of cross-linker, whose dipole moment drastically changed as T increased. Furthermore, the absence for these samples of the AP at ~(1596,1596) cm^−1^, previously observed for CNS (0.2:1), suggested that the aforementioned –COO^−^ and N–H groups were now conformationally stacked in their original state at 250 K, due to a change in the reticulated 3D structure.

**Figure 6:**
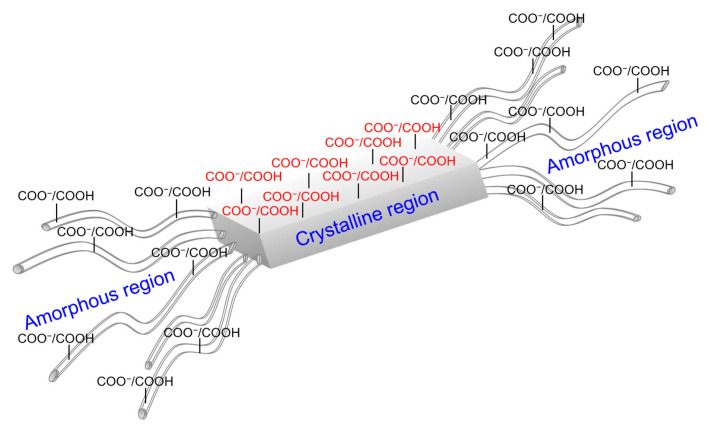
Schematic representation of the crystalline and amorphous domains of TEMPO-oxidized cellulose nano-fibers. Black and red COO^−^/COOH account for carboxyl functional groups of amorphous and crystalline regions, respectively. In this drawing, the bPEI segments were intentionally omitted.

A new scenario emerges by looking at the ACMs calculated for CNS (1:1) and CNS (2:1) systems (Figure 5h,i). Both the asynchronous correlation spectra resolved several highly overlapped peaks, as can be seen by the presence, in the 2D correlation plane of each sample, of two positive CPs at ~(1654,1583) cm^−1^ and at ~(1654,1635) cm^−1^, not revealed for CNS (0.2:1) (Figure 5g). In particular, as far as the first CP is concerned, we could associate its two coordinates to the (C=O)_amidic_ and (C=O)_carboxylic_ stretching vibrations belonging to the amide groups (1654 cm^−1^) and to the residual carboxylic groups (1583 cm^−1^) of the TEMPO-oxidized nano-cellulose chains. As a matter of fact, (C=O)_carboxylic_ group should be considered as free of inter-molecular linkages, not being involved in amide covalent interactions. Accordingly, the aforementioned mode reasonably arose from amorphous regions of the 3D polymer network, characterized by a low-tangle chains orientation. On the contrary, (C=O)_amidic_ stretching vibration reasonably came from crystalline domains in the xerogel phase, where the formation of amide bonds activated by the presence of bPEI took place. Interestingly, since Ω(1654,1583) > 0, as a main result we found that the (C=O)_carboxylic_ groups changed conformation, upon heating, after the (C=O)_amidic_ ones. This occurrence can be interpreted thinking that COO^–^ groups exhibits ion–ion intermolecular interactions which are stronger if compared to those developed by amide groups (–CO–NH–). The latter, being free of residual charge, can establish only lower-energy connections (such as hydrogen bonds) with the surrounding electronegative atoms, enhancing its susceptibility to thermal motion. Taking these considerations into account, the application of an external perturbation first affected the low-energy vibrations associated to the (C=O)_amidic_ groups, modifying their conformation, followed by the (C=O)_carboxylic_ ones, less susceptible to local re-organization. In addition, it is worth remarking that, as evidenced by the shape of the corresponding CPs (black stars in Figure 5h,i), the presence of different populations of both (C=O)_amidic_ and (C=O)_carboxylic_ functional groups of CNFs, involved in different molecular environments, could be clearly observed. In more detail, a careful inspection of Figure 5h,i reveals a “squeeze” of the corresponding CPs towards the vertical direction (in the figure, regions contoured by black dashed lines), as well as the onset of weak positive new CPs. This occurrence suggested that, keeping fixed the $\nu_{1}$ value at 1654 cm^−1^, several asynchronous cross correlations could be found in the 1583–1570 cm^−1^ range, which reasonably accounted for a collection of COO^−^ and COOH stretching vibrations characterized by a variety of electrostatic and steric surroundings, depending on the degree of crystallinity of a specific micro/nano domain of the polymer matrix. Considering the experimental procedure adopted for the synthesis of the bPEI/TOUS-CNFs sponges, surface COO^−^/COOH groups of the CNS crystalline nano-structures, and intra-chain COO^−^/COOH located in amorphous regions of the reticulated polymer network (see Figure 6) were present. As already reported [58,59], the C=O stretching vibration was strongly sensitive to the surrounding electrostatic environment, since a reduction of the co-operativity experienced by C=O groups resulted in a reinforcement of the associated dipole moment, leading to a shift of the corresponding stretching vibration frequency towards higher values. Accordingly, high-wavenumber modes reasonably arose from C=O oscillators in less constrained regions having low co-ordination degree, typical of amorphous moieties. On the contrary, low-wavenumber modes could be related to surface C=O oscillators of crystalline nano-structures, developing more a intense association.

Based on the aforementioned considerations, the inspection of Figure 5h,i indicates that a temperature increase led first to an initial re-arrangement of the amorphous COO^−^/COOH moieties, because of their high mobility due to the less constrained surrounding. After that, the energy transferred to the structure through thermal motion was able to modify the vibrational dynamics of the COO^−^/COOH groups in the crystalline region, characterized by higher co-operativity with respect to amorphous COO^−^/COOH.

In the case of CNS (0.2:1), the absence of the above discussed CP at ~(1654,1583) cm^−1^ revealed that such modes remained almost uncorrelated during the applied temperature trajectory, probably due to the low number of cellulose chain–chain interactions which enhanced cross-linking density at the expenses of the mobility of both functional groups. 

Concerning the CP at ~(1654,1635) cm^−1^ (blue stars in Figure 5h,i), the following sequence could be highlighted: peak at 1654 cm^−1^ change first, followed by peak at 1635 cm^−1^. The latter, not distinguishable in 1D FTIR-ATR conventional spectra, could be assigned to the HOH bending mode of absorbed water molecules into the polymer matrix coming from natural humidity. From this result, it turned out that the amidic carbonyl groups were affected by heating before the hydroxyl groups of absorbed water molecules. This occurrence could be associated, as evidenced in a previous work by the same authors [21], to the nano-confinement experienced by water molecules through the establishment of new physical cross-links (HBs). In particular, the presence of nano-sized cavities originating from the interlacing of bPEI and cellulose nano-fibers, and their size evolution upon hydration, was indirectly evidenced starting from the analysis of the so-called “short-range correlation length” [21]. The observed nanoscopic structural features in these systems may act as “shield” against external perturbations, heating in our case, resulting in a delayed effect of temperature on the HOH vibrational mode, thanks to the establishment of localized water “pockets”. In the case of CNS (0.2:1), no CP at ~(1654,1635) cm^−1^ was observed, suggesting that the external perturbation affects both vibrations in-phase, and therefore no chronological succession of events is highlighted. This occurrence can be explained hypothesizing a not fully-interlaced network, reasonably due to the low number of amino groups available for reticulation. As a result, water molecules are not affected by any shielding effect against thermal heating, and a simultaneous response of both the (C=O)_amidic_ and HOH bending mode takes place. 

The other observable correlations (in the figure, region contoured by black dotted lines) do not give any additional relevant information regarding the dynamical behavior exhibited by the investigated structure, and hence they won’t be discussed.

A 2DCoS analysis of the high frequency region (2680–3780 cm^−1^) was finally carried out in order to highlight the temporal evolution of the dynamical changes in the HB scheme of our bPEI/TOUS-CNFs materials, as T increased. In addition, a description of the sequential order of events affecting the N–H stretching mode of both primary and secondary amines will be given. About this, it is worth remarking that the effect of temperature on the HB network in polymer composites, such as multi-component hydrogels [60], sponge-like materials [61], and inclusion complexes [62], was already extensively investigated through FTIR-ATR spectroscopy. Variations of the HBs cooperativity degree upon the increase of temperature were evidenced, suggesting the activation of destructuring processes on the hydrogen bond pattern induced by thermal motion. Nevertheless, few experimental studies are currently available [63] aimed at providing the time sequence of events affecting both inter- and intrachain HBs (as well as the dynamics of high-energy modes) developing in complex materials. The knowledge of this additional aspect would get an insight into the overall conformational evolution in the polymer network induced by temperature, in turn related to the physico-chemical/mechanical properties, such as solubility and stiffness, exhibited by the resulting material [64,65,66]. In the light of the aforementioned considerations, the analysis of the high-frequency region will be limited to a detailed description of the ACMs only, aimed at achieving the step-by-step sequence affecting the high-frequency molecular motions.

**Figure 7:**
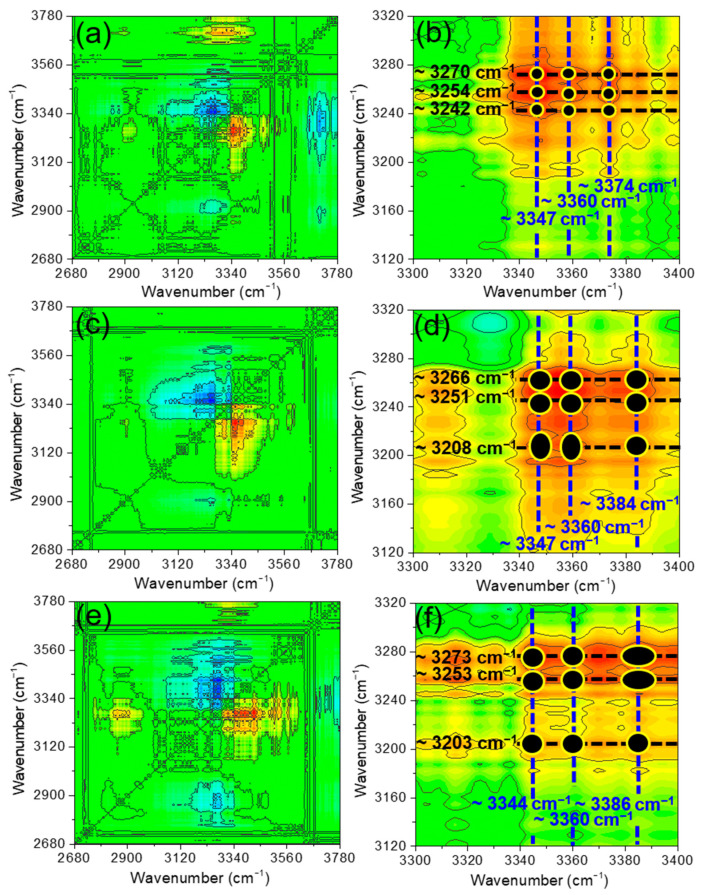
Calculated ACMs in the 2680–3780 cm^−1^ wavenumber range for CNS (0.2:1) (**a**), CNS (1:1) (**c**) and CNS (2:1) (**e**). For each of the investigated samples, a detailed view ((**b**) for CNS (0.2:1), (**d**) for CNS (1:1), and (**f**) for CNS (2:1)) of the (*v*_1_: 3330–3400 cm^−1^, *v*_2_: 3120–3320 cm^−1^) range, where most of the intra- and inter-chains correlations involving different population of OH groups of the polymer network falls, is also reported. Red, green and blue-colored areas describe positive, null and negative correlation intensities, respectively. Yellow countered black symbols account for regions characterized by maximum *asynchronous* intensity.

In Figure 7 we display the ACMs for all the investigated samples in the 2680–3780 cm^−1^ wavenumber range, and over the 250 K–340 K temperature range (250 K used as reference). 

**Figure 8:**
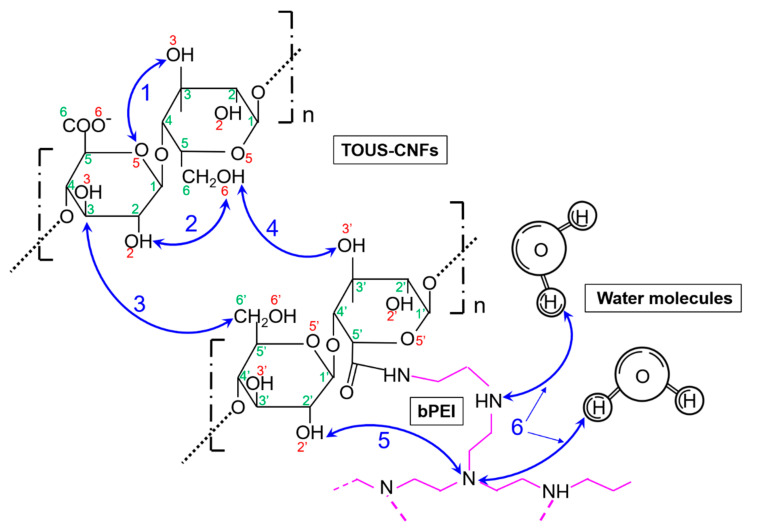
Schematic representation of the bPEI/TOUS-CNFs structure. Green and red numbers account for positions of carbon and oxygen atoms, respectively. Blue lines represent some of the major intra- (1 and 2, respectively associated to the O(3)H…O(5) and O(2)H…O(6)) and inter-chain (3 and 4, respectively associated to O(3)H…O(6′) and O(6)H…O(3′)) hydrogen bonds occurring in the cellulosic material. In addition, HBs of type 5 and 6 account for physical cross-links between primary/secondary amines of bPEI and H atoms belonging to CNFs or adsorbed water molecules, respectively. The reported figure has to be intended just as a fast visualization of the HBs network developed by the system under investigation, so it does not reflect the real chain-chain conformation of the specimen.

As reported in literature [67,68,69], three classes of HBs could be distinguished: (i) HBs between atoms located within the same cellulose unit; (ii) HBs between neighbor cellulose chains; (iii) HBs formed between adsorbed water molecules and the polymer structure. Furthermore, in our case, in view of a complete description of the HBs scheme, the presence of the cross-linking agent and its dynamical evolution upon heating must also be taken into account. As a matter of fact, cross-linker led to a considerable increase of nitrogen atoms within the polymer network (coming from amino functional groups of bPEI chains), providing new active sites available for physical cross-links (Figure 8).

In the case of CNS (0.2:1) (Figure 7a), a positive asynchronous correlation region centered at ~(3350,3255) cm^−1^ could be observed. A careful inspection points out that this area was made up of several contributions, highly overlapped, associated to distinct populations of OH oscillators involved in different molecular environments (see Figure 7b), and hence characterized by different cooperativity. In particular, based on well-established assignments reported in literature [70,71,72], peaks at $\nu_{1}∼3347{\;\mathrm{cm}}^{-1}$, $\nu_{1}∼3360{\;\mathrm{cm}}^{-1}$ and $\nu_{1}∼3374{\;\mathrm{cm}}^{-1}$ could be ascribed, respectively, to the N-H stretching mode ($\nu_{1}∼3347{\;\mathrm{cm}}^{-1}$) of bonded amides in bPEI (through the establishment, for example, of physical HBs or chemical links with surroundings) [73], and to the cellulose O(3)H…O(5) intrachain HBs ($\nu_{1}∼3360{\;\mathrm{cm}}^{-1}$ and $\nu_{1}∼3374{\;\mathrm{cm}}^{-1}$) [63]. On the other side, peaks at $\nu_{2}∼3242{\;\mathrm{cm}}^{-1}$ and $\nu_{2}∼3254{\;\mathrm{cm}}^{-1}$ were assigned to O(6)H…O(3′) interchain (or to the energy-equivalent O(3)H…O(6′)) HBs connecting adjacent segments [74]. Finally, a peak at $\nu_{2}∼3270{\;\mathrm{cm}}^{-1}$ was ascribed to the establishment of O(2)H…O(6) intrachain HBs [63].

The observed CPs (yellow countered black symbols in Figure 7b) were arranged on a grid in the 2D-plane, indicating that all features at the aforementioned $\nu_{1}$ values experience the effect of perturbation (T) earlier if compared to those at $\nu_{2}$. Taking into account the Noda’s rules and considering that the corresponding SCM was always positive within the explored range (data not shown), we could hypothesize the following sequence of events: (i) temperature promoted first the breaking of O(3)H…O(5) intrachain linkages (low energy HBs), leading to dynamical variations of the corresponding secondary O(3)H vibrational mode; (ii) at the same time, a destructuring process of HBs involving strongly electronegative N atoms of primary and secondary amines of bPEI and, most likely, H atoms of adsorbed H_2_O molecules (type 6), could be reasonably hypothesized (in agreement with what already observed from the analysis of low-wavenumber regions); (iii) after that, variations in the arrangement of both cellulose O(2)H…O(6) intrachain (type 2, Figure 8) and O(6)H…O(3′)/O(3)H…O(6′) interchain (type 3/4, Figure 8) HBs took place, in agreement with the fact that these linkages were found to be the most stable HBs in cellulose fibers [75]. 

Worthy of note, the absence of CPs having $\nu_{1}\geq 3400{\;\mathrm{cm}}^{-1}$, typical of vibrations of OH groups involved in low-cooperativity/non-hydrogen-bonded arrangements, implied that the conformational changes of these groups developed in-phase with all the steps of the above-described sequential process. 

As far as the CNS (1:1) and CNS (2:1) samples are concerned (Figure 7c–f), the detected CPs appeared almost equal in position and intensity to those in CNS (0.2:1), suggesting a similar temporal evolution, upon heating, of the high-frequency molecular dynamics. However, slight changes could still be recognized, indicating a not-negligible influence of the bPEI amount on the time-step sequence affecting the HBs scheme. As a main result (Figure 7d,f), a shift towards higher wavenumber of CPs characterized by $\nu_{1}∼3374{\;\mathrm{cm}}^{-1}$ in CNS (0.2:1), whose position moved, on average, to $\nu_{1}∼3385{\;\mathrm{cm}}^{-1}$ in CNS (1:1) and CNS (2:1), was detected, associated to a weakening of the O(3)H…O(5) intrachain linkages. These new correlations, not observable (or, at least, negligible) in the case of CNS (0.2:1), could be explained thinking that the O(3)H…O(5) intrachain HBs experienced a reduced association as a consequence of an increase in the amount of N atoms (that favor the formation of cellulose fiber-fiber HBs). At the same time, a shift towards lower wavenumber of CPs at $\nu_{2}∼3242{\;\mathrm{cm}}^{-1}$ in CNS (0.2:1), whose position moved, on average, to $\nu_{2}∼3205{\;\mathrm{cm}}^{-1}$ in CNS (1:1) and CNS (2:1), was observed, related to OH modes of strongly bounded adsorbed water molecules [76]. This occurrence indicated the presence of strongly bounded H_2_O, presumably confined in the CNS nano-cavities, whose thermal susceptibility was delayed with respect to the surrounding polymer network. This hypothesis is supported by the fact that, as reported in a previous FTIR-ATR study on these systems [22], the existence of liquid-like arrangements (having a co-operativity degree less than 4) of water molecules below the ice crystallization temperature, furnishing evidence of a supercooled behavior of adsorbed H_2_O, was already pointed out through the observation of a not-negligible intensity of its HOH bending band at T = 250 K.

In agreement with the analysis of the ACMs for CNS (1:1) and CNS (2:1) in the 1500–1700 cm^−1^ wavenumber range (Figure 5h,i), the observed delay could be ascribed to a shielding effect caused by a bPEI-induced, cellulose segments re-organization, which led to the formation of localized water “pockets”. 

Interestingly, the absence of prominent cross-correlations falling at $\nu_{2}∼3204{\;\mathrm{cm}}^{-1}$ in the case of CNS (0.2:1) (see Figure 7b) reasonably reflected the absence of such confinement geometries within the system, indicating that most of the adsorbed water molecules arranged themselves outside the reticulated CNS network, becoming totally exposed to thermal stimuli. 

## 4. Conclusions

2D correlation spectroscopy analysis was here carried out on a novel class of cellulose-based materials, with the aim of highlighting the complex sequence of events affecting the functional groups of the polymeric network upon heating from 250 K to 340 K, through the analysis of the synchronous and asynchronous correlation maps, describing coincidental and out-of-phase spectral variations of the original dataset, respectively. In particular, low-(900–1200 cm^−1^), medium-(1500–1700 cm^−1^), and high-(2680–3780 cm^−1^) wavenumber ranges of the medium infrared region were described in detail, providing different, sometimes complementary, information for a detailed description of the observed dynamical scenario upon the applied upward temperature trajectory.

Functional groups of both cellulose nano-fibers and bPEI segments were found to undergo dynamical changes following a precise sequence upon heating, in turn related to their mobility and location within the reticulated network. In particular, two dynamical responses associated to the C=O groups, respectively related to amidic and carboxylic moieties of the CNS network, were distinguished. In addition, depending on the crystallinity degree of the micro/nano domains of the polymer matrix, a different dynamical behavior of COO^−^/COOH groups upon thermal stimuli was highlighted. Going on, the dynamical behavior of functional groups (OH and NH) involved in intra- and interchain HB scheme developed in the CNS system was found to change according to a precise time-dependent evolution. On the contrary, low-cooperativity/non-hydrogen-bonded groups were observed not to experience any chronological succession. Moreover, in the case of CNS (1:1) and CNS (2:1) samples, the formation of water “pockets” within nano-cavities arising from cellulose segments re-organization was evidenced, causing a delayed effect of temperature on the strongly confined adsorbed H_2_O molecules.

Finally, the effect of the addition of an ever-increasing amount of cross-linker was discussed, aimed at providing a complete overview of the structural characteristics of the investigated systems, in view of their possible application as heterogeneous catalysts for reactions in water solvent. Finally, this first investigation opens the way for similar analyses by changing other parameters, like pH, in order to better clarifying the role of different functional groups also for other applications, for example as contaminants sorbent systems for water remediation.

## Figures and Tables

**Table 1:** List of specimens under investigation and their formulation.

Sample	TOUS-CNFs (g)	bPEI (g)
CNS (0.2:1)	1	0.2
CNS (1:1)	1	1
CNS (2:1)	1	2

**Table 2:** Sequential order of changes for a couple of correlated vibrational modes according to Noda’s rules.

Sign of Θ(*v*_1_, *v*_2_) ^1^	Sign of Ω(*v*_1_, *v*_2_) ^1^	Events Order ^1^
>0	>0	*v*_1_ prior *v*_2_
>0	<0	*v*_2_ prior *v*_1_
<0	>0	*v*_2_ prior *v*_1_
<0	<0	*v*_1_ prior *v*_2_

^1^ Assuming that (*v*_1_ > *v*_2_).
